# Supplementary material for: Delirium in patients with COVID-19 treated in the intensive care unit
Source: PLoS One. 2023 Nov 13;18(11):e0289662. doi: 10.1371/journal.pone.0289662 (PMC10642827; doi:10.1371/journal.pone.0289662)
Supplement: S1 Checklist — (DOCX) [file pone.0289662.s001.docx]

STROBE Statement—checklist of items that should be included in reports of observational studies

|  | Item No. | Recommendation | Page  No. | Relevant text from manuscript |
| --- | --- | --- | --- | --- |
| **Title and abstract** | 1 | (*a*) Indicate the study’s design with a commonly used term in the title or the abstract | 2 | Lines (26-28); This retrospective single-center study aimed to investigate the risk factors for delirium in patients with coronavirus disease 2019 infection receiving treatment in an intensive care unit. |
|  |  | (*b*) Provide in the abstract an informative and balanced summary of what was done and what was found | 2 | Lines (24-42); Abstracts |
| Introduction | | | |  |
| Background/rationale | 2 | Explain the scientific background and rationale for the investigation being reported | 2 and 3 | Lines (44-60) Introduction |
| Objectives | 3 | State specific objectives, including any prespecified hypotheses | 3 | Line (59-60); Therefore, the present study aimed to identify risk factors associated with delirium development and to investigate preventable factors in patients with COVID-19 receiving treatment in the ICU. |
| Methods | | | |  |
| Study design | 4 | Present key elements of study design early in the paper | 3 | Lines (63-70); This retrospective single-center study was conducted from February 2021 to April 2022 and included a total of 111 patients aged >18 years who had COVID-19, as confirmed by a positive reverse transcription–polymerase chain reaction test result, who required oxygen therapy, and who were admitted to the ICU. |
| Setting | 5 | Describe the setting, locations, and relevant dates, including periods of recruitment, exposure, follow-up, and data collection | 3 and 4 | Line (62-89); Materials and methods |
| Participants | 6 | (*a*) *Cohort study*—Give the eligibility criteria, and the sources and methods of selection of participants. Describe methods of follow-up  *Case-control study*—Give the eligibility criteria, and the sources and methods of case ascertainment and control selection. Give the rationale for the choice of cases and controls  *Cross-sectional study*—Give the eligibility criteria, and the sources and methods of selection of participants | 3 and 4 | Line (62-89); Materials and methods |
|  |  | (*b*) *Cohort study*—For matched studies, give matching criteria and number of exposed and unexposed  *Case-control study*—For matched studies, give matching criteria and the number of controls per case | 3 and 4 | Line (62-89); Materials and methods |
| Variables | 7 | Clearly define all outcomes, exposures, predictors, potential confounders, and effect modifiers. Give diagnostic criteria, if applicable | 3 | Line (67–76); Materials and methods |
| Data sources/ measurement | 8* | For each variable of interest, give sources of data and details of methods of assessment (measurement). Describe comparability of assessment methods if there is more than one group | 3 | Line (70-85); The patients’ characteristics included sex, age, smoking status, body mass index, factors for the assessment of underlying medical conditions and severity (Acute Physiology and Chronic Health Evaluation II [APACHE II] score and Sequential Organ Failure Assessment [SOFA] score), medications (steroids, tocilizumab, neuromuscular [NM] blocker, analgesic, sedative agent, and low-molecular-weight heparin), oxygen therapy (mechanical ventilator support, continuous renal replacement therapy, ECMO, and prone position), and length of stay in the ICU. The relationship between the occurrence of delirium and related factors was analyzed. |
| Bias | 9 | Describe any efforts to address potential sources of bias | 11 | Line (174-180); Limitations |
| Study size | 10 | Explain how the study size was arrived at | 3-4 | Line (62-89); Materials and methods |

Continued on next page

| Quantitative variables | 11 | Explain how quantitative variables were handled in the analyses. If applicable, describe which groupings were chosen and why | 4 | Line (91-97); statistical analysis |
| --- | --- | --- | --- | --- |
| Statistical methods | 12 | (*a*) Describe all statistical methods, including those used to control for confounding | 4 | Line (91-97); statistical analysis |
|  |  | (*b*) Describe any methods used to examine subgroups and interactions | 4 | Line (91-97); statistical analysis |
|  |  | (*c*) Explain how missing data were addressed | N/A | There were no missing data |
|  |  | (*d*) *Cohort study*—If applicable, explain how loss to follow-up was addressed  *Case-control study*—If applicable, explain how matching of cases and controls was addressed  *Cross-sectional study*—If applicable, describe analytical methods taking account of sampling strategy | 4 | Line (91-97); statistical analysis |
|  |  | (*e*) Describe any sensitivity analyses | 4 | Line (91-97); statistical analysis |
| Results | | | | |
| Participants | 13* | (a) Report numbers of individuals at each stage of study—eg numbers potentially eligible, examined for eligibility, confirmed eligible, included in the study, completing follow-up, and analysed | N/A |  |
|  |  | (b) Give reasons for non-participation at each stage | N/A |  |
|  |  | (c) Consider use of a flow diagram | N/A |  |
| Descriptive data | 14* | (a) Give characteristics of study participants (eg demographic, clinical, social) and information on exposures and potential confounders | 5 and 6 | Line (99-120); Patient characteristics, Table 1 |
|  |  | (b) Indicate number of participants with missing data for each variable of interest | 5 and 6 |  |
|  |  | (c) *Cohort study*—Summarise follow-up time (eg, average and total amount) | 5 and 6 | Line (101 - 130); Overall, 111 patients (average age: 64.1 years) were treated for COVID-19 in the ICU. Of these patients, four died due to pneumonia exacerbation (n = 2), multi-organ failure (n = 1), and multiple stomach cancer metastases (n = 1) after discharge from the ICU. |
| Outcome data | 15* | *Cohort study*—Report numbers of outcome events or summary measures over time | 6 and 10 | Line (122- 151); analysis of risk factors for delirium, characteristics of patients with mechanical ventilator support |
|  |  | *Case-control study—*Report numbers in each exposure category, or summary measures of exposure | N/A |  |
|  |  | *Cross-sectional study—*Report numbers of outcome events or summary measures | N/A |  |
| Main results | 16 | (*a*) Give unadjusted estimates and, if applicable, confounder-adjusted estimates and their precision (eg, 95% confidence interval). Make clear which confounders were adjusted for and why they were included | 6 and 10 | Line (122-151); Analysis of risk factors for delirium |
|  |  | (*b*) Report category boundaries when continuous variables were categorized | 6 and 10 | Line (122-151); Analysis of risk factors for delirium |
|  |  | (*c*) If relevant, consider translating estimates of relative risk into absolute risk for a meaningful time period | 6 and 10 | Line (122-151); Analysis of risk factors for delirium |

Continued on next page

| Other analyses | 17 | Report other analyses done—eg analyses of subgroups and interactions, and sensitivity analyses | 9 and 10 | Line (144-151); characteristics of patients with mechanical ventilator support |
| --- | --- | --- | --- | --- |
| Discussion | | | | |
| Key results | 18 | Summarise key results with reference to study objectives | 10 and 11 | Line (153-181); Discussion |
| Limitations | 19 | Discuss limitations of the study, taking into account sources of potential bias or imprecision. Discuss both direction and magnitude of any potential bias | 11 | Line (174-181); Discussion |
| Interpretation | 20 | Give a cautious overall interpretation of results considering objectives, limitations, multiplicity of analyses, results from similar studies, and other relevant evidence | 10 and 11 | Line (153-181); Discussion |
| Generalisability | 21 | Discuss the generalisability (external validity) of the study results | 10 and 11 | Line (153-181); Discussion |
| Other information | |  | | |
| Funding | 22 | Give the source of funding and the role of the funders for the present study and, if applicable, for the original study on which the present article is based |  | Submitted along with the online application form |

*Give information separately for cases and controls in case-control studies and, if applicable, for exposed and unexposed groups in cohort and cross-sectional studies.

**Note:** An Explanation and Elaboration article discusses each checklist item and gives methodological background and published examples of transparent reporting. The STROBE checklist is best used in conjunction with this article (freely available on the Web sites of PLoS Medicine at http://www.plosmedicine.org/, Annals of Internal Medicine at http://www.annals.org/, and Epidemiology at http://www.epidem.com/). Information on the STROBE Initiative is available at www.strobe-statement.org.
