# Supplementary material for: Delirium in patients with COVID-19 treated in the intensive care unit
Source: PLoS One. 2023 Nov 13;18(11):e0289662. doi: 10.1371/journal.pone.0289662 (PMC10642827; doi:10.1371/journal.pone.0289662)
Supplement: Attachment — Submitted filename: Response_Letter.docx [file pone.0289662.s003.docx]

**Response to Reviewer’s Comments**

Reviewer(s)' Comments to Author:

There is lack of evidence to support the development of X. Why choose N as sample size? The author should add baseline analysis of different type of SRBC to adjust the differences.

1. Data Interpretation: While the study identifies risk factors for delirium, it would be beneficial to provide more in-depth interpretation of the clinical implications of these findings. How can this knowledge be applied in clinical practice to prevent and manage delirium in COVID-19 patients?

**Reply:**

We would like to thank the Reviewer for the time and effort spent reviewing our manuscript and for providing comments, which have helped us improve our manuscript. We have made revisions based on your comments and have provided our point-by-point responses below. We hope that our responses and revisions appropriately address your comments.

A description has been added after the *Study limitations* paragraph of the discussion section (please see page 18).

This study identified the following risk factors for delirium in patients receiving ICU treatment for COVID-19 infection: old age, ICU stay, hypertension, mechanical ventilator support, and NM blockers. Thus, when an elderly COVID patient with hypertension is admitted to the ICU, the amount of sedative agent and NM blocker used to maintain ventilator care should be minimized, and a protocol should be established for ventilator-weaning to prevent delirium (e.g., daily spontaneous breathing trials).

2. Discussion of Sedative Agents: The manuscript mentions sedative agent administration as a risk factor for delirium. Further discussion on the types and dosages of sedative agents used, as well as their potential impact on delirium, would enhance the discussion section.

**Reply:**

Based on your comment, a description has been added to the study limitations paragraph of the discussion section (please see page 18).

In this study, 81.82% of patients used benzodiazepine as a sedative, and 90.91% used dexmedetomidine. Although benzodiazepines were used in relatively high doses per hour in patients with delirium, there was no statistical correlation (1.3±1.3 vs. 2.5±3.3, P=0.142). Relatively small doses of dexmedetomidine were used in patients with delirium, but there was no statistical correlation (0.3±0.2 vs. 0.2±0.2, P=0.388). Patients requiring ventilator treatment owing to COVID-19 infection often require relatively high doses of sedatives to maintain sedation [41]. This study also required a high dose to maintain sedation; therefore, a combination of benzodiazepine and dexmedetomidine was often necessary. Thus, unlike previous studies, there was no difference in the occurrence of delirium depending on the type of sedative agents.

3. Follow-up Data: The manuscript mentions that follow-up data after discharge from the ICU were not available. Discussing the importance of follow-up and potential directions for future research in this area could add depth to the discussion.

**Reply:**

Unfortunately, in the early stages of COVID-19 infection in Korea, patients were divided into those who were severe cases requiring oxygen treatment and those who did not require this treatment due a mild presentation. Treatment for the severe group was then referred to hospitals that had the capacity to do so. Once patients’ severe symptoms were cured, they were immediately transferred to a specialized hospital that treats mild symptoms. This was a single-center study; therefore, there was no follow-up of patients at the end of treatment. Although some patients could be followed up at the hospital where the study was conducted, most continued their remaining treatment at another hospital after transferring.

We completely agree with the reviewer that further research would help track patients treated at our hospital.

4. Clarity of Figures and Tables: Ensure that figures and tables are clear and well-labeled for readers to easily interpret the data presented.

**Reply:**

According to your comments, we have made some changes to ensure that the subsections for the categories are more distinguishable using bold writing, making the information clearer to the reader. We have removed any blank/empty cells and have formatted the tables to ensure that the text is presented in a clear manner.

5. Language and Grammar: While the manuscript is generally well-written, a final proofreading pass for language and grammar errors would enhance its professionalism.

**Reply:**

The manuscript has now undergone a final proofreading as per your suggestion.

6. Minor Comments about the Language

In the abstract, the sentence "Old age, intensive care unit stay, hypertension, mechanical ventilator support, and neuromuscular blocker use were predictive factors for the occurrence of delirium in patients with coronavirus disease 2019 infection receiving treatment in the intensive care unit" is quite long and could be broken down into shorter, more concise sentences for improved readability

**Reply:**

We have revised the sentence as per the reviewer’s suggestion to enhance conciseness, clarity, and overall readability (please see page 2):

In conclusion, old age, ICU stay, hypertension, mechanical ventilator support, and neuromuscular blocker use were predictive factors for delirium in COVID-19 patients in the ICU.
